# (4,4′-Dimethyl-2,2′-bipyridine-κ^2^ *N*,*N*′)(dimethyl sulfoxide-κ*O*)diiodidozinc(II)

**DOI:** 10.1107/S1600536810046763

**Published:** 2010-11-17

**Authors:** Mohammad Yousefi

**Affiliations:** a Islamic Azad University, Shahr-e-Rey Branch, Tehran, Iran

## Abstract

In the title compound, [ZnI_2_(C_12_H_12_N_2_)(C_2_H_6_OS)], the Zn^II^ ion is coordinated by two N atoms from a 4,4′-dimethyl-2,2′-bipyridine ligand, one O atom from a dimethyl sulfoxide mol­ecule and two I atoms in a distorted trigonal-bipyramidal geometry. Intra­molecular C—H⋯O hydrogen bonds and inter­molecular π–π stacking inter­actions between the pyridine rings [centroid–centroid distances = 3.637 (4) and 3.818 (4) Å] are present in the crystal structure.

## Related literature

For metal complexes of 4,4′-dimethyl-2,2′-bipyridine, see: Ahmadi *et al.* (2008 ▶); Alizadeh *et al.* (2010 ▶); Amani *et al.* (2009 ▶); Bellusci *et al.* (2008 ▶); Hojjat Kashani *et al.* (2008 ▶); Kalateh *et al.* (2008 ▶, 2010 ▶); Sakamoto *et al.* (2004 ▶); Sofetis *et al.* (2006 ▶); Willett *et al.* (2001 ▶); Yoshikawa *et al.* (2003 ▶); Yousefi *et al.* (2008 ▶).
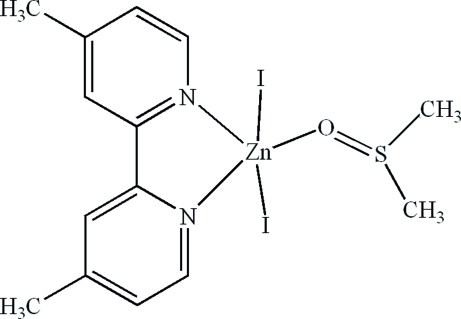


## Experimental

### 

#### Crystal data

[ZnI_2_(C_12_H_12_N_2_)(C_2_H_6_OS)]
*M* *_r_* = 581.56
Monoclinic, 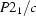

*a* = 8.6173 (7) Å
*b* = 15.5424 (11) Å
*c* = 14.8976 (10) Å
β = 102.908 (6)°
*V* = 1944.9 (3) Å^3^
*Z* = 4
Mo *K*α radiation
μ = 4.54 mm^−1^
*T* = 298 K
0.40 × 0.20 × 0.10 mm

#### Data collection

Bruker APEX CCD diffractometer
Absorption correction: multi-scan (*SADABS*; Sheldrick, 1996 ▶) *T*_min_ = 0.350, *T*_max_ = 0.636
21034 measured reflections
5243 independent reflections
4246 reflections with *I* > 2σ(*I*)
*R*_int_ = 0.113

#### Refinement

*R*[*F* ^2^ > 2σ(*F* ^2^)] = 0.076
*wR*(*F* ^2^) = 0.202
*S* = 1.20
5243 reflections
191 parameters
H-atom parameters constrained
Δρ_max_ = 2.11 e Å^−3^
Δρ_min_ = −2.87 e Å^−3^

### 

Data collection: *SMART* (Bruker, 2007 ▶); cell refinement: *SAINT* (Bruker, 2007 ▶); data reduction: *SAINT*; program(s) used to solve structure: *SHELXTL* (Sheldrick, 2008 ▶); program(s) used to refine structure: *SHELXTL*; molecular graphics: *ORTEP-3* (Farrugia, 1997 ▶); software used to prepare material for publication: *WinGX* (Farrugia, 1999 ▶).

## Supplementary Material

Crystal structure: contains datablocks I. DOI: 10.1107/S1600536810046763/hy2375sup1.cif

Structure factors: contains datablocks I. DOI: 10.1107/S1600536810046763/hy2375Isup2.hkl

Additional supplementary materials:  crystallographic information; 3D view; checkCIF report

## Figures and Tables

**Table 1:** Selected bond lengths (Å)

Zn1—N1	2.135 (5)
Zn1—N2	2.167 (6)
Zn1—O1	2.112 (5)
Zn1—I1	2.6199 (9)
Zn1—I2	2.6944 (9)

**Table 2:** Hydrogen-bond geometry (Å, °)

*D*—H⋯*A*	*D*—H	H⋯*A*	*D*⋯*A*	*D*—H⋯*A*
C1—H1⋯O1	0.93	2.41	2.938 (9)	116

**Fig. 1.**
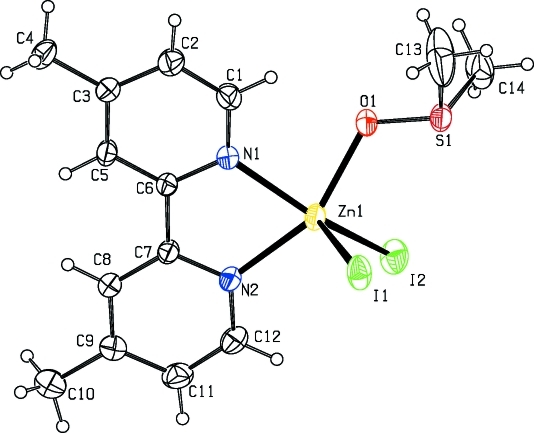
The molecular structure of the title compound. Displacement ellipsoids are drawn at the 30% probability level.

**Fig. 2.**
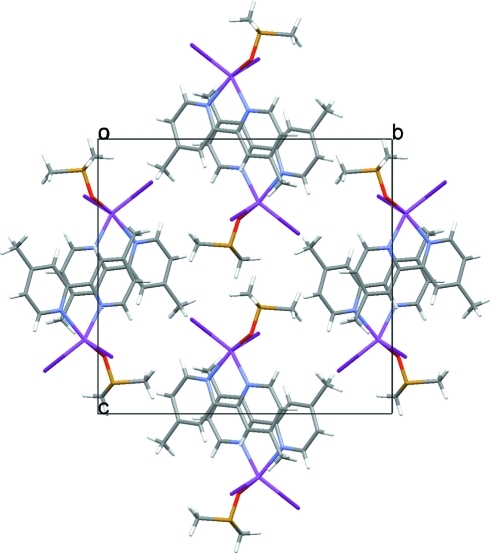
Crystal packing diagram for the title compound.

## supplementary crystallographic information

### Comment

4,4'-Dimethyl-2,2'-bipyridine (4,4'-dmbipy) is a good bidentate ligand and numerous complexes with 4,4'-dmbipy have been prepared, such as that of mercury (Kalateh *et al.*, 2008; Yousefi *et al.*, 2008), indium (Ahmadi *et al.*, 2008), iron (Amani *et al.*, 2009), platinum (Hojjat Kashani *et al.*, 2008), manganese (Sakamoto *et al.*, 2004), silver (Bellusci *et al.*, 2008), gallium (Sofetis *et al.*, 2006), copper (Willett *et al.*, 2001), zinc (Alizadeh *et al.*, 2010), cadmium (Kalateh *et al.*, 2010) and iridium (Yoshikawa *et al.*, 2003). Here, we report the synthesis and structure of the title compound.

In the title compound (Fig. 1), the Zn^II^ ion is coordinated by two N atoms from a 4,4'-dmbipy ligand, one O atom from a dimethyl sulfoxide molecule and two I^-^ anions in a distorted trigonal-bipyramidal geometry. The Zn—I, Zn—O and Zn—N bond lengths are collected in Table 1. In the crystal structure, intramolecular C—H···O hydrogen bonds (Table 2) and intermolecular π–π stacking interactions (Fig. 2) between the pyridine rings, *Cg*2···*Cg*2^i^ and *Cg*2···*Cg*3^ii^ [symmetry codes: (i) 1-x, -y, 1-z; (ii) -x, -y, 1-z; *Cg*2 and *Cg*3 are the centroids of the N1, C1, C2, C3, C5, C6 ring and N2, C7, C8, C9, C11, C12 ring, respectively] may stabilize the structure, with centroid–centroid distances of 3.637 (4) and 3.818 (4) Å.

### Experimental

For the preparation of the title compound, a solution of 4,4'-dmbipy (0.15 g, 0.80 mmol) in methanol (10 ml) was added to a solution of ZnI_2_ (0.25 g, 0.80 mmol) in methanol (5 ml) at room temperature. Crystals suitable for X-ray diffraction experiment were obtained by methanol diffusion into a colorless solution in DMSO after one week (yield: 0.34 g, 73.1%).

### Refinement

H atoms were positioned geometrically and refined as riding atoms, with C—H = 0.93 (aromatic) and 0.96 (methyl) Å and with *U*_iso_(H) = 1.2*U*_eq_(C). The highest residual electron density was found 0.81 Å from I2 and the deepest hole 0.86 Å from I1.

### Crystal data

**Table:**

[ZnI_2_(C_12_H_12_N_2_)(C_2_H_6_OS)]	*F*(000) = 1104
*M**_r_* = 581.56	*D*_x_ = 1.986 Mg m^−^^3^
Monoclinic, *P*2_1_/*c*	Mo *K*α radiation, λ = 0.71073 Å
Hall symbol: -P 2ybc	Cell parameters from 21034 reflections
*a* = 8.6173 (7) Å	θ = 2.4–29.2°
*b* = 15.5424 (11) Å	µ = 4.54 mm^−^^1^
*c* = 14.8976 (10) Å	*T* = 298 K
β = 102.908 (6)°	Block, colorless
*V* = 1944.9 (3) Å^3^	0.40 × 0.20 × 0.10 mm
*Z* = 4	

### Data collection

**Table:**

Bruker APEX CCD diffractometer	5243 independent reflections
Radiation source: fine-focus sealed tube	4246 reflections with *I* > 2σ(*I*)
graphite	*R*_int_ = 0.113
φ and ω scans	θ_max_ = 29.2°, θ_min_ = 2.4°
Absorption correction: multi-scan (*SADABS*; Sheldrick, 1996)	*h* = −11→11
*T*_min_ = 0.350, *T*_max_ = 0.636	*k* = −21→21
21034 measured reflections	*l* = −20→19

### Refinement

**Table:**

Refinement on *F*^2^	Primary atom site location: structure-invariant direct methods
Least-squares matrix: full	Secondary atom site location: difference Fourier map
*R*[*F*^2^ > 2σ(*F*^2^)] = 0.076	Hydrogen site location: inferred from neighbouring sites
*wR*(*F*^2^) = 0.202	H-atom parameters constrained
*S* = 1.20	*w* = 1/[σ^2^(*F*_o_^2^) + (0.1105*P*)^2^ + 1.119*P*] where *P* = (*F*_o_^2^ + 2*F*_c_^2^)/3
5243 reflections	(Δ/σ)_max_ = 0.006
191 parameters	Δρ_max_ = 2.11 e Å^−^^3^
0 restraints	Δρ_min_ = −2.87 e Å^−^^3^

### Fractional atomic coordinates and isotropic or equivalent isotropic displacement parameters (Å^2^)

**Table:**

	*x*	*y*	*z*	*U*_iso_*/*U*_eq_	
C1	0.3959 (9)	0.0849 (4)	0.5994 (5)	0.0499 (15)	
H1	0.4491	0.1110	0.6537	0.060*	
C2	0.4035 (10)	0.1229 (5)	0.5175 (5)	0.0558 (18)	
H2	0.4613	0.1734	0.5171	0.067*	
C3	0.3249 (8)	0.0859 (4)	0.4355 (5)	0.0434 (13)	
C4	0.3297 (12)	0.1243 (6)	0.3445 (5)	0.060 (2)	
H4C	0.2999	0.0816	0.2972	0.072*	
H4B	0.4356	0.1442	0.3456	0.072*	
H4A	0.2568	0.1718	0.3320	0.072*	
C5	0.2391 (8)	0.0114 (4)	0.4415 (4)	0.0404 (12)	
H5	0.1819	−0.0146	0.3880	0.049*	
C6	0.2382 (7)	−0.0246 (4)	0.5263 (4)	0.0348 (11)	
C7	0.1490 (7)	−0.1048 (4)	0.5361 (4)	0.0371 (11)	
C8	0.0708 (8)	−0.1531 (5)	0.4614 (4)	0.0444 (13)	
H8	0.0759	−0.1363	0.4022	0.053*	
C9	−0.0137 (9)	−0.2252 (5)	0.4731 (5)	0.0520 (16)	
C10	−0.0965 (13)	−0.2786 (6)	0.3928 (7)	0.072 (2)	
H10C	−0.1468	−0.3269	0.4146	0.087*	
H10B	−0.0202	−0.2987	0.3595	0.087*	
H10A	−0.1755	−0.2443	0.3528	0.087*	
C11	−0.0155 (10)	−0.2477 (5)	0.5633 (6)	0.0578 (18)	
H11	−0.0723	−0.2957	0.5749	0.069*	
C12	0.0667 (11)	−0.1988 (5)	0.6351 (5)	0.0563 (18)	
H12	0.0660	−0.2157	0.6949	0.068*	
C13	0.573 (3)	0.1599 (8)	0.8800 (9)	0.148 (9)	
H13A	0.4826	0.1882	0.8428	0.178*	
H13B	0.6632	0.1665	0.8523	0.178*	
H13C	0.5975	0.1849	0.9404	0.178*	
C14	0.7238 (12)	0.0134 (10)	0.9372 (7)	0.093 (4)	
H14A	0.7977	0.0373	0.9044	0.112*	
H14B	0.7258	−0.0482	0.9334	0.112*	
H14C	0.7531	0.0307	1.0006	0.112*	
N1	0.3148 (6)	0.0114 (3)	0.6051 (3)	0.0390 (10)	
N2	0.1480 (7)	−0.1279 (4)	0.6227 (3)	0.0408 (11)	
O1	0.4955 (6)	0.0209 (4)	0.7891 (3)	0.0512 (11)	
Zn1	0.28482 (9)	−0.04667 (5)	0.72994 (5)	0.0401 (2)	
I1	0.07682 (6)	0.04536 (4)	0.79226 (3)	0.05824 (19)	
I2	0.37974 (8)	−0.18094 (4)	0.84331 (4)	0.0642 (2)	
S1	0.5317 (2)	0.05061 (13)	0.88831 (11)	0.0482 (4)	

### Atomic displacement parameters (Å^2^)

**Table:**

	*U*^11^	*U*^22^	*U*^33^	*U*^12^	*U*^13^	*U*^23^
C1	0.057 (4)	0.047 (3)	0.043 (3)	−0.012 (3)	0.005 (3)	−0.002 (3)
C2	0.070 (5)	0.052 (4)	0.046 (4)	−0.016 (3)	0.013 (3)	0.003 (3)
C3	0.045 (3)	0.046 (3)	0.039 (3)	−0.005 (3)	0.010 (2)	0.005 (2)
C4	0.079 (5)	0.064 (4)	0.042 (4)	−0.011 (4)	0.021 (4)	0.012 (3)
C5	0.046 (3)	0.049 (3)	0.027 (2)	0.001 (3)	0.009 (2)	0.000 (2)
C6	0.035 (3)	0.040 (3)	0.030 (2)	−0.001 (2)	0.009 (2)	−0.002 (2)
C7	0.038 (3)	0.044 (3)	0.030 (3)	0.001 (2)	0.009 (2)	0.004 (2)
C8	0.046 (3)	0.052 (3)	0.036 (3)	−0.009 (3)	0.012 (2)	−0.001 (3)
C9	0.050 (4)	0.057 (4)	0.049 (4)	−0.012 (3)	0.009 (3)	0.001 (3)
C10	0.075 (6)	0.073 (5)	0.068 (5)	−0.025 (5)	0.014 (4)	−0.009 (4)
C11	0.058 (4)	0.052 (4)	0.064 (5)	−0.010 (3)	0.016 (3)	0.012 (3)
C12	0.065 (5)	0.057 (4)	0.048 (4)	−0.007 (3)	0.014 (3)	0.018 (3)
C13	0.29 (3)	0.070 (7)	0.058 (6)	−0.031 (11)	−0.023 (10)	−0.004 (5)
C14	0.048 (4)	0.171 (12)	0.055 (5)	0.020 (6)	0.000 (4)	−0.011 (7)
N1	0.038 (2)	0.046 (3)	0.032 (2)	−0.004 (2)	0.0059 (19)	0.000 (2)
N2	0.041 (3)	0.051 (3)	0.031 (2)	−0.002 (2)	0.0088 (19)	0.006 (2)
O1	0.045 (2)	0.078 (3)	0.030 (2)	−0.008 (2)	0.0059 (18)	−0.003 (2)
Zn1	0.0420 (4)	0.0511 (4)	0.0273 (3)	0.0043 (3)	0.0079 (3)	0.0021 (3)
I1	0.0498 (3)	0.0841 (4)	0.0397 (3)	0.0204 (2)	0.00761 (19)	−0.0118 (2)
I2	0.0731 (4)	0.0624 (3)	0.0512 (3)	0.0118 (2)	0.0009 (2)	0.0195 (2)
S1	0.0445 (8)	0.0693 (11)	0.0297 (7)	0.0026 (7)	0.0062 (6)	0.0014 (6)

### Geometric parameters (Å, °)

**Table:**

C1—N1	1.352 (9)	C10—H10B	0.9600
C1—C2	1.370 (10)	C10—H10A	0.9600
C1—H1	0.9300	C11—C12	1.372 (12)
C2—C3	1.383 (10)	C11—H11	0.9300
C2—H2	0.9300	C12—N2	1.341 (9)
C3—C5	1.388 (9)	C12—H12	0.9300
C3—C4	1.490 (9)	C13—S1	1.746 (12)
C4—H4C	0.9600	C13—H13A	0.9600
C4—H4B	0.9600	C13—H13B	0.9600
C4—H4A	0.9600	C13—H13C	0.9600
C5—C6	1.384 (8)	C14—S1	1.751 (10)
C5—H5	0.9300	C14—H14A	0.9600
C6—N1	1.334 (7)	C14—H14B	0.9600
C6—C7	1.488 (8)	C14—H14C	0.9600
C7—N2	1.341 (7)	Zn1—N1	2.135 (5)
C7—C8	1.387 (9)	Zn1—N2	2.167 (6)
C8—C9	1.368 (10)	O1—S1	1.513 (5)
C8—H8	0.9300	Zn1—O1	2.112 (5)
C9—C11	1.392 (11)	Zn1—I1	2.6199 (9)
C9—C10	1.498 (12)	Zn1—I2	2.6944 (9)
C10—H10C	0.9600		
			
N1—C1—C2	123.3 (7)	C12—C11—H11	120.0
N1—C1—H1	118.3	C9—C11—H11	120.0
C2—C1—H1	118.3	N2—C12—C11	122.8 (7)
C1—C2—C3	119.7 (7)	N2—C12—H12	118.6
C1—C2—H2	120.2	C11—C12—H12	118.6
C3—C2—H2	120.2	S1—C13—H13A	109.5
C2—C3—C5	117.0 (6)	S1—C13—H13B	109.5
C2—C3—C4	122.0 (6)	H13A—C13—H13B	109.5
C5—C3—C4	121.1 (6)	S1—C13—H13C	109.5
C3—C4—H4C	109.5	H13A—C13—H13C	109.5
C3—C4—H4B	109.5	H13B—C13—H13C	109.5
H4C—C4—H4B	109.5	S1—C14—H14A	109.5
C3—C4—H4A	109.5	S1—C14—H14B	109.5
H4C—C4—H4A	109.5	H14A—C14—H14B	109.5
H4B—C4—H4A	109.5	S1—C14—H14C	109.5
C6—C5—C3	120.5 (6)	H14A—C14—H14C	109.5
C6—C5—H5	119.7	H14B—C14—H14C	109.5
C3—C5—H5	119.7	C6—N1—C1	117.4 (5)
N1—C6—C5	122.1 (6)	C6—N1—Zn1	117.2 (4)
N1—C6—C7	115.5 (5)	C1—N1—Zn1	125.2 (4)
C5—C6—C7	122.4 (5)	C12—N2—C7	117.9 (6)
N2—C7—C8	121.4 (6)	C12—N2—Zn1	126.3 (5)
N2—C7—C6	115.7 (5)	C7—N2—Zn1	115.7 (4)
C8—C7—C6	123.0 (5)	S1—O1—Zn1	122.0 (3)
C9—C8—C7	121.3 (6)	O1—Zn1—N1	83.89 (19)
C9—C8—H8	119.4	O1—Zn1—N2	150.80 (19)
C7—C8—H8	119.4	N1—Zn1—N2	75.84 (19)
C8—C9—C11	116.7 (7)	O1—Zn1—I1	99.96 (15)
C8—C9—C10	121.6 (7)	N1—Zn1—I1	107.68 (15)
C11—C9—C10	121.8 (7)	N2—Zn1—I1	106.06 (15)
C9—C10—H10C	109.5	O1—Zn1—I2	90.64 (15)
C9—C10—H10B	109.5	N1—Zn1—I2	142.63 (15)
H10C—C10—H10B	109.5	N2—Zn1—I2	92.86 (14)
C9—C10—H10A	109.5	I1—Zn1—I2	109.67 (3)
H10C—C10—H10A	109.5	O1—S1—C13	103.2 (5)
H10B—C10—H10A	109.5	O1—S1—C14	106.0 (4)
C12—C11—C9	119.9 (7)	C13—S1—C14	99.2 (9)
			
N1—C1—C2—C3	0.3 (13)	C8—C7—N2—C12	−1.0 (10)
C1—C2—C3—C5	0.9 (12)	C6—C7—N2—C12	178.8 (6)
C1—C2—C3—C4	−179.7 (8)	C8—C7—N2—Zn1	177.7 (5)
C2—C3—C5—C6	−1.9 (10)	C6—C7—N2—Zn1	−2.6 (7)
C4—C3—C5—C6	178.7 (7)	S1—O1—Zn1—N1	151.4 (4)
C3—C5—C6—N1	1.8 (10)	S1—O1—Zn1—N2	−162.7 (3)
C3—C5—C6—C7	−179.5 (6)	S1—O1—Zn1—I1	44.4 (4)
N1—C6—C7—N2	4.2 (8)	S1—O1—Zn1—I2	−65.7 (4)
C5—C6—C7—N2	−174.5 (6)	C6—N1—Zn1—O1	160.7 (5)
N1—C6—C7—C8	−176.1 (6)	C1—N1—Zn1—O1	−24.2 (6)
C5—C6—C7—C8	5.2 (10)	C6—N1—Zn1—N2	1.9 (4)
N2—C7—C8—C9	1.6 (11)	C1—N1—Zn1—N2	177.0 (6)
C6—C7—C8—C9	−178.1 (7)	C6—N1—Zn1—I1	−100.8 (4)
C7—C8—C9—C11	−0.6 (12)	C1—N1—Zn1—I1	74.3 (6)
C7—C8—C9—C10	−179.1 (8)	C6—N1—Zn1—I2	77.7 (5)
C8—C9—C11—C12	−0.8 (13)	C1—N1—Zn1—I2	−107.2 (6)
C10—C9—C11—C12	177.6 (9)	C12—N2—Zn1—O1	131.6 (6)
C9—C11—C12—N2	1.5 (14)	C7—N2—Zn1—O1	−46.9 (7)
C5—C6—N1—C1	−0.5 (9)	C12—N2—Zn1—N1	179.0 (7)
C7—C6—N1—C1	−179.3 (6)	C7—N2—Zn1—N1	0.5 (4)
C5—C6—N1—Zn1	174.9 (5)	C12—N2—Zn1—I1	−76.3 (6)
C7—C6—N1—Zn1	−3.8 (7)	C7—N2—Zn1—I1	105.2 (4)
C2—C1—N1—C6	−0.5 (11)	C12—N2—Zn1—I2	35.1 (6)
C2—C1—N1—Zn1	−175.6 (6)	C7—N2—Zn1—I2	−143.4 (4)
C11—C12—N2—C7	−0.5 (12)	Zn1—O1—S1—C13	−127.7 (9)
C11—C12—N2—Zn1	−179.0 (6)	Zn1—O1—S1—C14	128.5 (6)

### Hydrogen-bond geometry (Å, °)

**Table:**

*D*—H···*A*	*D*—H	H···*A*	*D*···*A*	*D*—H···*A*
C1—H1···O1	0.93	2.41	2.938 (9)	116
